# Differential effects of DEAE negative mode chromatography and gel-filtration chromatography on the charge status of *Helicobacter pylori* neutrophil-activating protein

**DOI:** 10.1371/journal.pone.0173632

**Published:** 2017-03-22

**Authors:** Zhi-Wei Hong, Yu-Chi Yang, Timothy Pan, Huey-Fen Tzeng, Hua-Wen Fu

**Affiliations:** 1 Institute of Molecular and Cellular Biology, National Tsing Hua University, Hsinchu, Taiwan, Republic of China; 2 Department of Life Science, National Tsing Hua University, Hsinchu, Taiwan, Republic of China; 3 Department of Applied Chemistry, National Chi Nan University, Puli, Nantou, Taiwan, Republic of China; Instituto Butantan, BRAZIL

## Abstract

*Helicobacter pylori* neutrophil-activating protein (HP-NAP) is involved in *H*. *pylori*-associated gastric inflammation. HP-NAP is also a vaccine candidate, a possible drug target, and a potential diagnostic marker for *H*. *pylori*-associated diseases. Previously, we purified recombinant HP-NAP by one-step diethylaminoethyl (DEAE) negative mode chromatography by collecting the unbound fraction at pH 8.0 at 4°C. It remains unclear why HP-NAP does not bind to DEAE resins at the pH above its isoelectric point during the purification. To investigate how pH affects the surface net charge of HP-NAP and its binding to DEAE resins during the purification, recombinant HP-NAP expressed in *Escherichia coli* was subjected to DEAE negative mode chromatography at pH ranging from 7.0 to 9.0 at 25°C and the surface charge of purified HP-NAP was determined by capillary electrophoresis. A minimal amount of HP-NAP was detected in the elution fraction of DEAE Sepharose resin at pH 8.5, whereas recombinant HP-NAP was detected in the elution fraction of DEAE Sephadex resin only at pH 7.0 and 8.0. The purified recombinant HP-NAP obtained from the unbound fractions was not able to bind to DEAE resins at pH 7.0 to 9.0. In addition, the surface charge of the purified HP-NAP was neutral at pH 7.0 to 8.0 and was either neutral or slightly negative at pH 8.5 and 9.0. However, recombinant HP-NAP purified from gel-filtration chromatography was able to bind to DEAE Sepharose resin at pH 7.0 to 9.0 and DEAE Sephadex resin at pH 7.0. At pH 8.5 and 9.0, only the negatively charged species of HP-NAP were found. Thus, recombinant HP-NAP with different charge status can be differentially purified by DEAE negative mode chromatography and gel-filtration chromatography. Furthermore, the charge distribution on the surface of HP-NAP, the presence of impure proteins, and the overall net charge of the resins all affect the binding of HP-NAP to DEAE resins during the negative purification.

## Introduction

*Helicobacter pylori* (*H*. *pylori*) is a Gram-negative, microaerophilic pathogen, which colonizes the gastric mucosa of human stomach. The bacteria infect at least half of the global population [1]. *H*. *pylori* infection is recognized as a major cause of chronic gastritis and peptic ulcer and considered as a risk factor of gastric cancer [2]. The gastric mucosa infected by *H*. *pylori* typically shows a massive infiltration of neutrophils [3, 4]. *Helicobacter pylori* neutrophil-activating protein (HP-NAP), a chemotactic factor of neutrophils, may play a key role in the pathogenesis of *H*. *pylori*-induced gastric inflammation.

HP-NAP was first identified in the water extract of *H*. *pylori* for its ability to promote neutrophil adhesion to endothelial cells and production of reactive oxygen species (ROS) by neutrophils [5]. This protein is also capable of activating neutrophils, monocytes and mast cells to secrete pro-inflammatory cytokines and inflammatory mediators [6–10], which could further activate gastric inflammation and result in the damage of gastric mucosa. In addition, HP-NAP promotes T helper type 1 (Th1) polarization responses [7]. This protein drives the maturation of dendritic cells to enable them to induce a Th1-type cytokine-dominant profile and antigen-specific T cell proliferation [11]. High amounts of tumor necrosis factor alpha (TNF-α) and interferon gamma (IFN-γ) were found to be produced by antigen-specific gastric Th cells upon HP-NAP stimulation [7]. Thus, both innate and adaptive immune responses induced by HP-NAP contribute to the pathological outcome during *H*. *pylori* infection.

HP-NAP is a spherical protein formed by twelve identical subunits [12, 13]. Each subunit is a four-α-helix bundle protein with a molecular mass of approximately 17 kDa [12, 13]. Dodecameric HP-NAP is about 9–10 nm in diameter and has a negatively charged internal cavity for storage of iron [12, 13]. The structure of HP-NAP is similar to those of DNA-protecting proteins from starved cells (Dps) and Dps-related ferritin [12]. HP-NAP is capable of binding up to 500 iron atoms and displays ferroxidase activity *in vitro* [12]. Two ferroxidase centers (FOCs) are located at the interface formed between the helices 1 and 2 from each HP-NAP monomer [13, 14]. However, only one iron ion was observed in each ferroxidase center in the crystal structures of HP-NAP [13, 14]. In comparison of the apo-structure of HP-NAP, binding of the iron ion causes significant conformational changes of the residues (Trp26, Asp52, and Glu56) located at the FOC, which further induces the displacement of helix 2 [14, 15]. Since the conformational changes of these resides and the displacement of helix 2 occur close to and at the interior side of the central cavity of HP-NAP, HP-NAP should still keep its spherical shell-like structure upon iron binding. However, the spherical shell-like dodecamer of HP-NAP might reduce its size upon iron binding as suggested by a structural analysis of zinc-substituted HP-NAP showing that its diameter is much shorter than that of apo-HP-NAP [15, 16]. Unlike the other Dps-like proteins or ferritin, HP-NAP has a large number of positively charged amino acid residues on its surface [13]. The prevalence of positively charged residues present on the surface of HP-NAP suggests that HP-NAP could play a role in neutrophil activation similar to some chemokines [17, 18]. It has been proposed that the helix 3 (Leu69-Leu75), the helix 4 (Lys89-Leu114), or the linking coils (His63-Thr68 and Thr76-Ser88) of HP-NAP are critical in stimulating neutrophil activation [19]. Interestingly, all the positively charged residues located at these regions are exposed on the surface of HP-NAP. These surface-exposed positively charged residues are most likely involved in HP-NAP-induced activation of neutrophils.

Despite its pathogenic role, HP-NAP has potential clinical applications due to its immunogenic and immunomodulatory properties. HP-NAP can be used for vaccine development, clinical diagnosis, and drug development [20]. Several approaches have been developed to purify recombinant HP-NAP in its native form [19, 21, 22]. Among these approaches, at least two chromatographic steps, including a final gel filtration step, are needed to obtain HP-NAP with high purity. Recently, we have developed a one-step diethylaminoethyl (DEAE) negative mode chromatography to purify native recombinant HP-NAP overexpressed in *Escherichia coli* (*E*. *coli*) with high purity and high yield by collecting the unbound fraction at pH 8.0 [23, 24]. During the purification at 4°C, a minimal amount of HP-NAP was detected in the elution fraction at pH 8.0, whereas a substantial amount of HP-NAP was present in the elution at pH 7.0 and 9.0 [23]. Although the isoelectric point of HP-NAP has reported to be 6.75 as determined by isoeletrofocusing [12], most of the recombinant HP-NAP bound to DEAE resins at pH 7.0 but not at pH 8.0 during the purification at 4°C from either *E*. *coli* or *B*. *subtilis* [23, 25]. The reason for this finding remains unclear. In this work, we have investigated whether HP-NAP binds to DEAE resins during and after its purification at pH ranging from 7.0 to 9.0 at 25°C. Capillary electrophoresis was also utilized to examine the surface charge of HP-NAP purified from DEAE negative mode chromatography at the pH investigated. For comparison, similar experimental approaches were applied to characterize HP-NAP purified from gel-filtration chromatography to further evaluate its surface charge and ability to bind to DEAE resins.

## Materials and methods

### Expression of recombinant HP-NAP in *E*. *coli*

The *napA* gene [GenBank:AE000543.1, Gene: HP0243] was amplified from genomic DNA of *H*. *pylori* strain 26695 (Genbank Accession number AE000543) (accession no. AE000543, ATCC) and then cloned into pET42a expression vector as previously described [22]. The plasmid pET42a-NAP was transformed into *E*. *coli* BL21(DE3). The expression of HP-NAP in *E*. *coli* was induced with 0.4 mM isopropyl β-D-1-thiogalactopyranoside (IPTG) as described previously [20]. The *E*. *coli* pellet was stored at -70°C until use.

### Bacterial cell lysis

For purification of recombinant HP-NAP by DEAE negative mode batch chromatography, the pellet harvested from a 9 ml culture of *E*. *coli* expressing HP-NAP was resuspended in one-third of the culture volume of ice-cold buffer containing 20 mM Tris-HCl and 50 mM NaCl (pH 9.0 at 25°C) plus 0.1% (v/v) protease inhibitor mixture (PI mix). The PI mix contained 0.13 M phenylmethylsulfonyl fluoride (PMSF), 0.03 M N-alpha-tosyl-L-lysyl-chloromethyl ketone (TLCK), and 0.03 M N-tosyl-L-phenylalanyl-chloromethyl ketone (TPCK). The bacterial suspensions were disrupted by an ultrasonic processor SONICS VCX-750 (Sonics & Materials, Newtown, CT, USA) on ice at 20% amplitude with 1 sec on and 1 sec off pulses for 5 min. The cell lysate was centrifuged at 30,000 x *g* at 4°C for 1 h to separate soluble protein fraction and insoluble debris. For purification of HP-NAP by gel-filtration chromatography, the pellet harvested from a 300 ml culture of *E*. *coli* expressing HP-NAP was resuspended in one-fifteenth of the culture volume of ice-cold buffer containing 20 mM Tris-HCl and 50 mM NaCl (pH 9.0 at 25°C) plus 0.1% (v/v) PI mix. The bacterial suspension was disrupted by EmulsiFlex-C3 high pressure homogenizer (Avestin Inc., Ottawa, Canada) at 17,000 psi for 7 passes. The cell lysate was centrifuged at 30,000 x *g* at 4°C for 1 h to separate soluble protein fraction and insoluble debris.

### pH adjustment

The pH of the soluble protein fraction of *E*. *coli* lysate and HP-NAP purified from gel- filtration chromatography was kept at pH 9.0 or adjusted to pH 8.5, 8.0, 7.5, and 7.0 at 25°C with the addition of HCl. The pH adjustment of the protein solutions was all carried out at 4°C. To achieve the desired pH at 25°C, 1 ml of the ice-cold protein solutions at pH 9.0 (25°C) was added with an appropriate amount of HCl with a concentration less or equal to 2 N and with a volume less than 1% of the total volume. These protein solutions were then added with an appropriate amount of the ice-cold buffer containing 20 mM Tris-HCl and 50 mM NaCl at their respective pH values (25°C) to keep the same protein concentration for each protein solution.

### Purification of recombinant HP-NAP by DEAE negative mode batch chromatography

The soluble protein fraction of *E*. *coli* expressing HP-NAP at pH 9.0 and those adjusted to pH 8.5, 8.0, 7.5, and 7.0 were subjected to a small-scale DEAE negative mode batch chromatography with DEAE Sephadex A-25 (Sigma-Aldrich, St. Louis, MO, USA) and DEAE Sepharose fast flow (Amersham Pharmacia Biotech, Uppsala, Sweden) resins at their respective pH values as previously described [23] except that the purification was performed at 25°C. The protein concentration of the soluble protein fraction was 0.3 mg/ml. The volume ratio of soluble protein fraction to resin was 3:1. The unbound fraction, wash fraction, and elution fraction were obtained by collecting the supernatant after centrifugation as previously described [23]. These fractions collected were analyzed by SDS-PAGE, native-PAGE, and immunoblotting. The unbound fraction, which contained the purified HP-NAP, was stored at 4°C before further measurement.

### Purification of HP-NAP by gel-filtration chromatography

Recombinant HP-NAP from a 5 ml of the soluble protein fraction of *E*. *coli* expressing HP-NAP was purified by two consecutive gel filtration steps using an XK 16/100 column packed with Sephacryl S-300 high resolution resin (Sephacryl S-300 HR) (GE Healthcare Bio-Sciences, Pittsburgh, PA, USA) and a HiLoad 16/60 Superdex 200 prep grade (Superdex 200 pg) gel filtration column (GE Healthcare Bio-Sciences) at 4°C as previously described [22] except that the buffer containing 20 mM Tris-HCl and 50 mM NaCl (pH 9.0 at 25°C) was used in both gel filtration steps. After each step of purification, fractions containing HP-NAP were analyzed by SDS-PAGE and native-PAGE. The peak fractions containing HP-NAP, from the second gel filtration step, were pooled and stored at 4°C for no more than one month. A volume of 200 μl of standard proteins used for gel filtration (Bio‐Rad, Hercules, CA, USA) was applied to the Superdex 200 pg gel filtration column to estimate the molecular weight of recombinant HP‐NAP.

### Examination of the binding ability of purified HP-NAP to DEAE resins

The unbound fractions obtained from DEAE negative mode batch chromatography at pH 9.0, 8.5, 8.0, 7.5, and 7.0 at 25°C were loaded onto DEAE Sephadex and DEAE Sepharose resins pre-equilibrated with 20 mM Tris-HCl and 50 mM NaCl at their respective pH at 25°C. The volume ratio of unbound fraction to resin was 3:1. The unbound fraction, wash fraction, and elution fraction were obtained by collecting the supernatant after centrifugation as described for purification of recombinant HP-NAP by DEAE negative mode batch chromatography. The fractions including the unbound fraction, wash fraction, and elution fraction were analyzed by SDS-PAGE.

The recombinant HP-NAP purified from gel-filtration chromatography was kept at pH 9.0 or adjusted to pH 8.5, 8.0, 7.5, and 7.0 at 25°C and then adjusted to a protein concentration of 0.3 mg/ml. The protein samples were loaded onto DEAE Sephadex and DEAE Sepharose resins pre-equilibrated with the buffer containing 20 mM Tris-HCl and 50 mM NaCl at pH 9.0, 8.5, 8.0, 7.5, and 7.0 at 25°C. The ratio of sample volume to resin volume was 3:1. The unbound fraction, wash fraction, and elution fraction were obtained as described for purification of recombinant HP-NAP by DEAE negative mode batch chromatography and analyzed by SDS-PAGE.

### Immunoblotting

Immunoblotting was performed essentially the same as previously described [23]. SDS-PAGE-separated proteins were transferred onto a polyvinylidene difluoride (PVDF) membrane. The membrane was blocked with 5% nonfat milk in Tris-buffered saline/Tween-20 (TBST) containing 50 mM Tris‐HCl, pH 7.4, 15 mM NaCl, and 0.1% Tween‐20 at room temperature for 1 h. The membrane was then probed with hybridoma culture supernatants containing mouse monoclonal antibody MAb 16F4 against HP-NAP [26] with a dilution factor of 1:200 in TBST containing 5% bovine serum albumin (BSA) at 4°C overnight. The membrane was washed three times with 5% nonfat milk/TBST, for 10 min each time, and then probed with horseradish peroxidase-conjugated anti-mouse secondary antibody (Jackson ImmunoResearch, West Grove, PA, USA) at a dilution of 1:5000 in 5% nonfat milk/TBST at room temperature for 1 h. After the membrane was washed three times with TBST for 10 min each, the signal of HP-NAP on the membrane was visualized by an enhanced chemiluminescence (ECL) system (PerkinElmer, Waltham, MA, USA) and detected by LAS-3000 imaging system (Fujifilm, Tokyo, Japan).

### Capillary electrophoresis

Capillary electrophoresis (CE) was carried out using Agilent Technologies CE system (Agilent Technologies, Wilmington, DE, USA), equipped with a diode array detector (DAD) with a wavelength range of 192 to 600 nm and controlled by Chemstation. CE was performed in an uncoated fused-silica capillary of 48.5 cm (40 cm effective length, the length from the injection end to the detector) x 75 μm I.D. (Polymicro Technologies, Phoenix, AZ, USA) and detected by the on-column measurement of UV absorption at 220 nm. Measurements were conducted at 12 kV for at least 7 min at 25°C. 4-Methoxybenzyl alcohol (4mBA) at a concentration of 0.1 mM was used as the neutral marker to calculate the electroosmotic flow (EOF). HP-NAP used for analysis included (i) the recombinant HP-NAP obtained from the unbound fractions from DEAE negative mode batch chromatography at pH 7.0, 7.5, 8.0, 8.5, and 9.0 from the two resins and (ii) the recombinant HP-NAP purified from gel-filtration chromatography at pH 9.0 (25°C) and those ones with further pH adjustment to pH 8.5, 8.0, 7.5 and 7.0. The samples composed of HP-NAP alone or with the addition of 4mBA were hydrodynamically injected at 50 mbar for 5 sec. Data were collected and processed by Chemstation. Prior to the first use, the new capillary was rinsed with 1 M NaOH for 5 min and then with distilled water for 10 min. Each separation was preceded by a 6-min rinse with 0.1 M NaOH, a 1.5-min rinse with distilled water, and a 3.5-min rinse with the running buffer. In order to avoid migration time drift, the buffer was replenished after each injection. The capillary was stored in water when not in use. The electrophoretic mobility (μ_e_) was calculated by the following equation:

$$\mu_{e}=\frac{L\times l}{V}\left(\frac{1}{t}-\frac{1}{t_{4mBA}}\right)$$

where *L* is the total length of the capillary, *l* is the length from the injection end to the detector, *V* is the applied voltage, and *t* and *t*_*4mBA*_ are the migration times of sample and 4mBA, the neutral EOF marker, respectively.

### Analytical ultracentrifugation

HP-NAP purified by gel-filtration chromatography was kept at pH 9.0 or adjusted to pH 8.5, 8.0, 7.5, or 7.0. The sedimentation coefficients of HP-NAP in the buffers containing 20 mM Tris-HCl and 50 mM NaCl at pH 9.0, 8.5, 8.0, 7.5, and 7.0 at 25°C were determined by analytical ultracentrifugation (AUC) with a PorteomeLab XL-I protein characterization system (Beckman Coulter, Brea, CA, USA) essentially as previously described [25] with some modifications. The sample and reference sectors were filled with 392 μl of HP-NAP at a concentration of 0.3 mg/ml and 424 μl of its reference buffer, respectively. Centrifugation was carried out at 38,000 rpm at a temperature of 25°C for at least 180 min. Absorbance data were collected at a wavelength of 280 nm with a time interval of 3 min and a radial increment of 0.003 cm. Sedimentation coefficient distribution, c(s), was analyzed by the software SEDFIT with appropriate buffer densities, buffer viscosities, and partial specific volumes of HP-NAP at 25°C as determined in SEDNTERP (http://sednterp.unh.edu/). Apparent molar mass values were calculated by SEDFIT.

### Circular dichroism spectroscopy

The recombinant HP-NAP purified from gel-filtration chromatography was kept at pH 9.0 or adjusted to pH 8.5, 8.0, 7.5, or 7.0 at 25°C. Secondary structures of HP-NAP in the buffers containing 20 mM Tris-HCl and 50 mM NaCl at pH 9.0, 8.5, 8.0, 7.5, and 7.0 at 25°C were assessed by circular dichroism (CD) spectroscopy on an Aviv model 62A DS CD spectrophotometer (Aviv Biomedical, Lakewood, NJ, USA) essentially as previously described [23] with some modification. Far-UV CD spectra were recorded for HP-NAP at a concentration of 0.3 mg/ml using a quartz cuvette with a 1 mm path length at 1 nm intervals between 195 and 260 nm at 25°C. An averaging time of 0.5 sec was utilized. The CD signals of each protein sample and its respective reference buffer from a total of three scans were averaged. The CD signal of recombinant HP-NAP was obtained by subtracting the CD signal of the reference buffer. The mean residue ellipticity (MRE) was calculated from the formula: MRE = θ/(10 × L × C × N), where θ is the CD signal (mdeg), L is the path length of the cuvette (cm), C is the molar concentration of the protein (M), and N is the number of peptide bonds.

### Miscellaneous methods

Protein concentrations were analyzed by the Bradford method [27] using a commercial dye preparation (Bio-Rad, Hercules, CA, USA), and BSA was used as a standard. SDS-PAGE and native-PAGE were performed as previously described [25] by using gels containing 15% and 10% acrylamide, respectively.

## Results

### Effect of pH on the binding of HP-NAP to DEAE resins in batch chromatography

**Fig 1:**
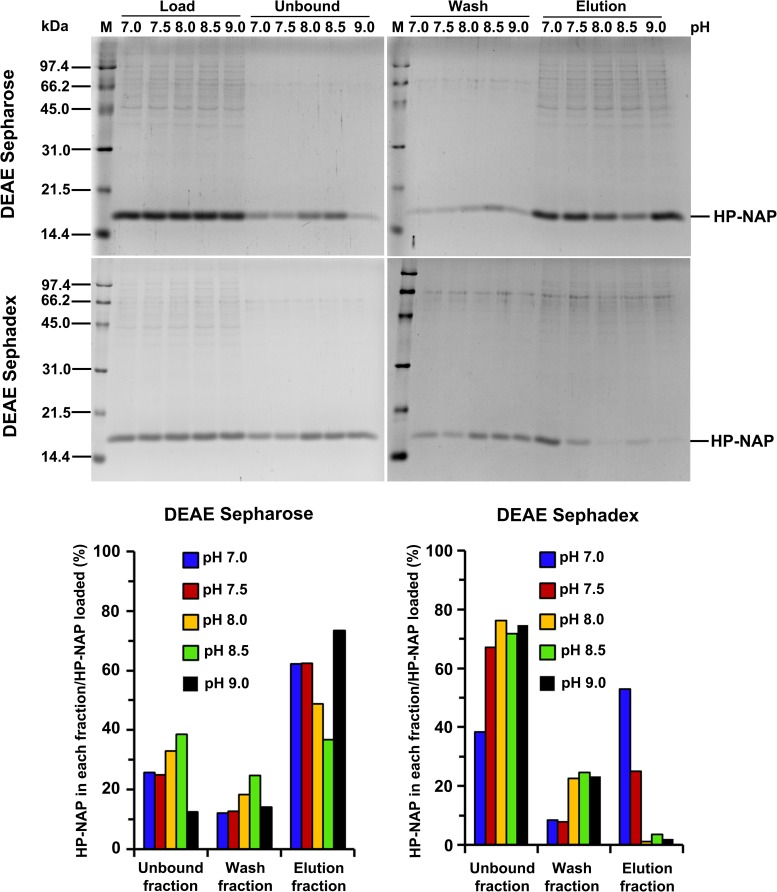
SDS-PAGE analysis of the purification of HP-NAP by DEAE negative mode chromatography at pH 7.0 to 9.0. The soluble protein fraction of *E*. *coli* BL21(DE3) expressing HP-NAP was adjusted to pH ranging from 7.0 to 9.0 at 25°C and then diluted to a protein concentration of 0.3 mg/ml. The samples were individually loaded onto DEAE Sepharose and DEAE Sephadex resins to purify recombinant HP-NAP by a batch method as described in Materials and Methods. The pH-adjusted soluble protein fractions, indicated as load, and the unbound, wash and elution fractions were analyzed by SDS-PAGE. Molecular masses (M) in kDa are indicated on the left of the stained gels and the blots. The percent ratio of the amount of recombinant HP-NAP detected in each fraction to the amount of HP-NAP loaded on the resin at each pH was calculated from the intensity of HP-NAP band on SDS gels for each fraction divided by the sum of those for the unbound, wash and elution fractions. Similar results were obtained from at least two independent experiments.

**Fig 2:**
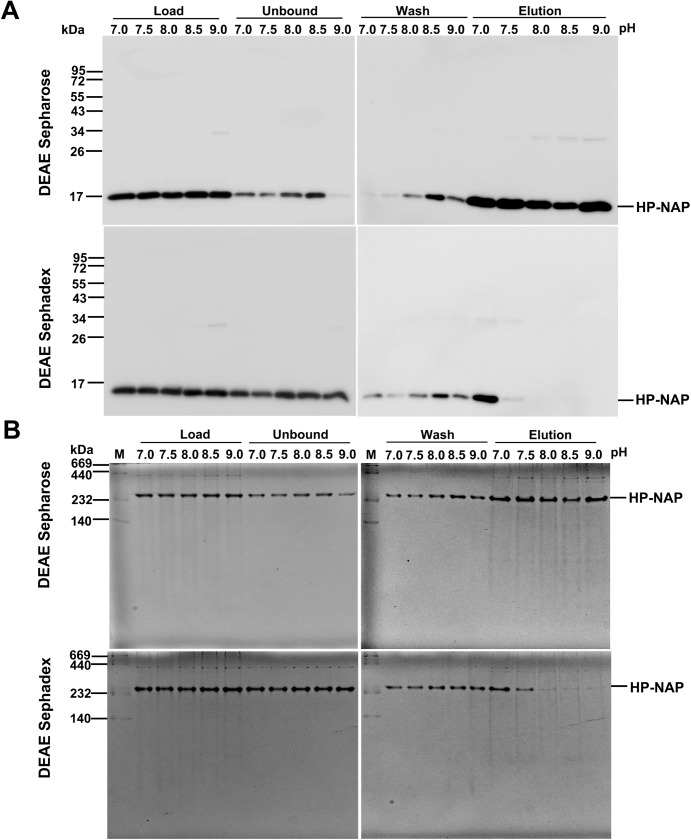
Immunoblot and native‐PAGE analysis of the purification of HP‐NAP by DEAE negative mode chromatography at pH 7.0 to 9.0. The protein samples are prepared the same as those described in Fig 1. The pH-adjusted soluble protein fractions, indicated as load, and the unbound, wash and elution fractions collected from DEAE Sephadex and DEAE Sepharose resins were analyzed by immunoblotting (**A**) and native‐PAGE (**B**). Molecular masses (M) in kDa are indicated on the left of the stained gels. Similar results were obtained from at least two independent experiments.

In our previous studies, we found that recombinant HP-NAP bound to DEAE anion-exchange resins at pH 7.0 but not at pH 8.0 during purification at 4°C from either *E*. *coli* or *B*. *subtilis* [23, 25]. However, this result is unexpected since the surface charge of HP-NAP should be more negative at pH 8.0 than at pH 7.0. Here, we reinvestigate whether HP-NAP binds to DEAE resins at pH ranging from 7.0 to 9.0 during the purification at 25°C. The recombinant HP-NAP expressed in *E*. *coli* was subjected to batch chromatography using DEAE Sepharose and DEAE Sephadex resins under these conditions. For DEAE Sepharose resin, at pH 7.0, the majority of recombinant HP-NAP was present in the elution fraction (Fig 1). The amount of recombinant HP-NAP present in the elution fraction decreased gradually with increasing pH up to 8.5 and then increased at pH 9.0 (Fig 1). At pH 8.5, the least amount of recombinant HP-NAP was detected in the elution fraction whereas a greater amount of HP-NAP was present in the unbound and wash fractions (Fig 1). For DEAE Sephadex resin, more than half of the recombinant HP-NAP was present in the elution fraction at pH 7.0 (Fig 1). The amount of recombinant HP-NAP in the elution fraction decreased gradually with increasing pH (Fig 1). At pH 8.0 to 9.0, recombinant HP-NAP was not detected in the elution fraction (Fig 2A) but was mostly present in the unbound and wash fractions (Figs 1 & 2A). A much higher binding ability of HP-NAP to DEAE Sepharose resin than to DEAE Sephadex resin was observed especially at pH 9.0 (Fig 1). In addition, the purity of HP-NAP in the unbound fractions at all pH values investigated was higher than 90% (Fig 1). The presence of the recombinant HP-NAP in each fraction was confirmed by immunoblot analysis using a monoclonal antibody against HP-NAP (Fig 2A). The recombinant HP-NAP present in each fraction was oligomeric with apparent molecular mass of around 232 kDa as analyzed by native-PAGE (Fig 2B). These results show that HP-NAP bound least to DEAE Sepharose resin at pH 8.5 and did not bind to DEAE Sephadex resin at pH ranging from pH 8.0 to 9.0 at 25°C. However, this finding is in conflict with the knowledge that more HP-NAP should bind to DEAE resins as pH increases since the net negative surface charge of HP-NAP increases as pH increases.

### Effect of surface charge and impure proteins on the interaction of HP-NAP and DEAE resins at pH 7.0 to 9.0

**Fig 3:**
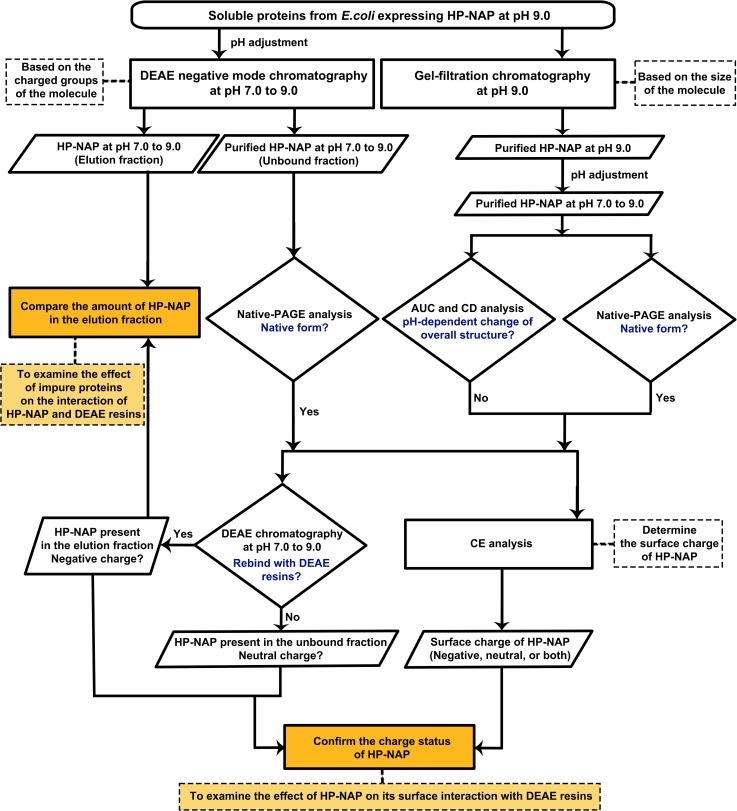
Schematic diagram of experimental approaches to investigate the effect of the surface charge of HP-NAP and impure proteins on the interaction of HP-NAP and DEAE resins.

**Fig 4:**
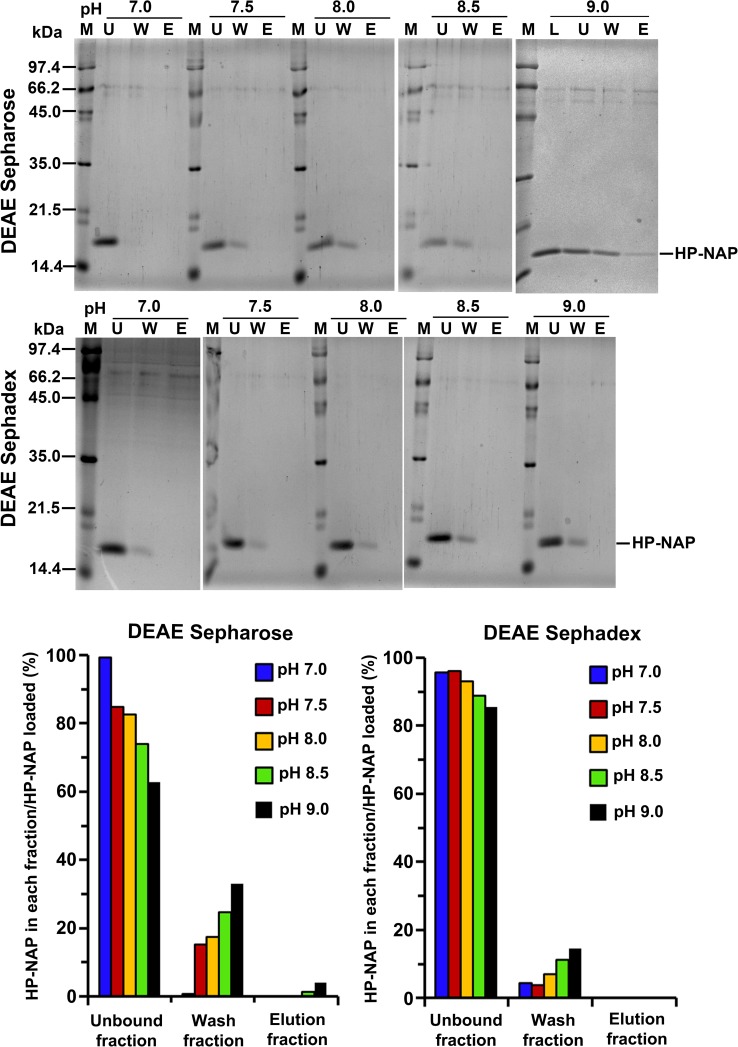
Binding ability of HP-NAP purified from DEAE negative mode chromatography to DEAE resins at pH 7.0 to 9.0. The purified recombinant HP-NAP obtained from the unbound fraction of DEAE Sepharose and DEAE Sephadex resins from the batch chromatography at pH 7.0 to 9.0 at 25°C was re-subjected to the batch chromatography under the same conditions as described in Materials and Methods to examine the binding ability of pure HP-NAP to the two DEAE resins. The unbound (U), wash (W) and elution (E) fractions were analyzed by SDS-PAGE on 15% gels. Molecular masses (M) in kDa are indicated on the stained gels. The percent ratio of the amount of recombinant HP-NAP detected in each fraction to the amount of HP-NAP loaded on the resin at each pH was calculated from the intensity of HP-NAP band on SDS gels for each fraction divided by the sum of those for the unbound, wash and elution fractions. Similar results were obtained from at least two to three independent experiments. Note: The purified recombinant HP-NAP obtained from the unbound fraction from DEAE negative mode chromatography at 9.0, indicated as load (L), is shown.

The surface charge of HP-NAP and impure proteins present in the crude cell extracts could be the main factors that contribute to the finding that the amount of recombinant HP-NAP binding to DEAE anion-exchange resins did not increase as pH was increased from 7.0 to 9.0. A flow chart summarizing the experimental approaches to investigate the effect of the surface change of HP-NAP and impure proteins on the interaction of HP-NAP and DEAE resins is shown in Fig 3. Here, we first determined whether an indirect interaction between HP-NAP and the resin occurs through other impure proteins present in the crude cell extract during purification. In this experiment, recombinant HP-NAP purified from the unbound fractions of both DEAE resins from batch chromatography was re-subjected to the DEAE batch chromatography under the same conditions to examine whether the pure recombinant HP-NAP binds to the DEAE resins. For both resins, recombinant HP-NAP purified from the unbound fractions was still present in the unbound fractions at pH ranging from 7.0 to 8.5 (Fig 4). At pH 9.0, recombinant HP-NAP purified from the unbound fraction was mostly detected in the unbound fraction for DEAE Sepharose resin but was completely present in the unbound fraction for DEAE Sephadex resin (Fig 4). Since pure recombinant HP-NAP does not bind to both DEAE resins at pH 7.0, the presence of more than half of the recombinant HP-NAP in the elution fractions at pH 7.0 from the batch chromatography for both DEAE resins shown in Fig 1 could be due to the presence of impure proteins during the purification. The results suggest that impure proteins assist the binding of recombinant HP-NAP to DEAE resins at pH 7.0 during the purification. The finding that recombinant HP-NAP is present in the unbound fractions for both DEAE resins at the pH values investigated suggests that neutral molecular species of HP-NAP were purified by DEAE negative mode chromatography at these different pH values. In addition, the partial binding of the recombinant HP-NAP purified from the unbound fraction at pH 9.0 to DEAE Sepharose resin indicates that a small amount of the recombinant HP-NAP purified from the unbound fraction at pH 9.0 is negatively charged.

**Fig 5:**
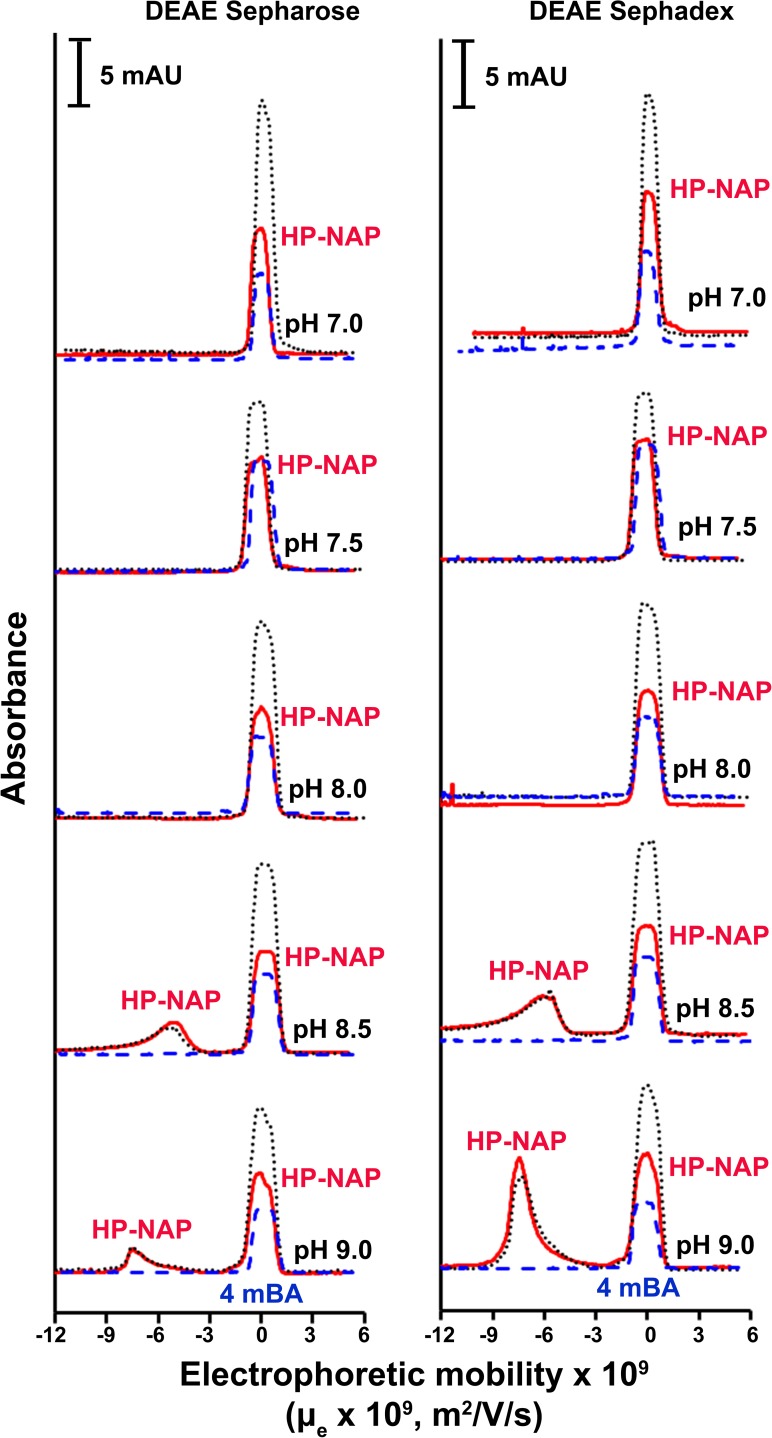
Capillary electropherograms of HP-NAP purified from DEAE negative mode chromatography at pH 7.0 to 9.0. The recombinant HP-NAP obtained from the unbound fractions of DEAE Sepharose and DEAE Sephadex resins at pH 7.0 to 9.0 was subjected to CE analysis at 25°C as described in Materials and Methods. The UV absorbance was recorded at 220 nm. 4-Methoxybenzyl alcohol (4mBA) was used as the neutral electroosmotic flow (EOF) marker, which migrated as a peak at μ_e_ = 0 for all electropherograms. The peaks shown in the electropherograms are HP-NAP (━), 4mBA (**—-**), and HP-NAP together with 4mBA (⋯). Data were representative of two independent experiments.

We next applied capillary electrophoresis (CE) to determine the surface charge of recombinant HP-NAP purified from the unbound fractions of both resins at pH ranging from 7.0 to 9.0 to investigate whether the surface charge of the pure recombinant HP-NAP plays a role on its interaction with DEAE resins. At pH 7.0 to 8.0, only one peak was detected and this peak was overlapping with the peak of the neutral electroosmotic flow (EOF) marker (Fig 5), indicating that the purified HP-NAP is neutral at pH 7.0 to 8.0. At pH 8.5 and 9.0, two peaks were detected with one overlapping with the peak of the neutral EOF marker and the other corresponding to the molecular species having negative electrophoretic mobility (Fig 5), indicating that both neutral and negatively charged molecular species of HP-NAP are present at pH 8.5 and 9.0. The electrophoretic mobility (μ_e_) of the negatively charged HP-NAP purified from DEAE Sepharose resin is -5.1 x 10^−9^ and -7.4 x 10^−9^ m^2^V^-1^s^-1^ at pH 8.5 and pH 9.0, respectively, and the electrophoretic mobility (μ_e_) of the negatively charged HP-NAP purified from DEAE Sephadex resin is -6.0 x 10^−9^ and -7.5 x 10^−9^ m^2^V^-1^s^-1^ at pH 8.5 and pH 9.0, respectively (Table 1). The greater the absolute value of μ_e_ at pH 9.0 as compared to that at pH 8.5 indicates that more negative charges are on the surface of the charged HP-NAP at pH 9.0 than at pH 8.5. However, at pH 9.0, the ratio of the negatively charged molecular species to the neutral molecular species of HP-NAP purified from DEAE Sepharose resin is much smaller than that of HP-NAP purified from DEAE Sephadex resin (Fig 5). This result suggests that during the purification at pH 9.0, the negatively charged HP-NAP binds to DEAE Sepharose resin but flows through DEAE Sephadex resin and the neutral HP-NAP flows through both resins. It is consistent with the finding that at pH 9.0, a small amount of the negatively charged recombinant HP-NAP purified from the unbound fraction of DEAE Sepharose resin can bind back to the resin (Fig 4). Taken together, recombinant HP-NAP purified from the unbound fractions of DEAE resins is neutral at pH 7.0 to 8.0 and is either neutral or negatively charged at pH 8.5 and 9.0.

**Table 1:** The proposed surface charge and the calculated electrophoretic mobility of HP-NAP purified by DEAE negative mode chromatography and gel-filtration chromatography at pH 7.0 to 9.0 based on the CE data.

	HP-NAP surface charge; HP-NAP electrophoretic mobility (m^2^/V/s) ^a^		
pH	DEAE negative mode chromatography ^b^		Gel-filtration chromatography ^c^
	DEAE sepharose	DEAE sephadex	
7.0	• neutral ^d^	• neutral ^d^	NA ^e^
7.5	• neutral ^d^	• neutral ^e^	NA ^e^
8.0	• neutral ^d^	• neutral ^d^	NA ^e^
8.5	• neutral ^f^ • negative;–(5.06 ± 0.03) x 10^−9^	• neutral ^f^ • negative;–(6.02 ± 0.09) x 10^−9^	• negative;–(5.36 ± 0.03) x 10^−9^
9.0	• neutral ^f^ • negative;–(7.47 ± 0.02) x 10^−9^	• neutral ^f^ • negative;–(7.40 ± 0.08) x 10^−9^	• negative;–(7.10 ± 0.10) x 10^−9^

^a^ Only the electrophoretic mobility of the negative species is shown.

^b^ The electropherograms of the CE experiments are shown in Fig 5.

^c^ The electropherograms of the CE experiments are shown in Fig 9.

^d^ All HP-NAP co-migrated with the neutral EOF marker, 4-methoxybenzyl alcohol as shown in Fig 5.

^e^ NA: not available. No peak was detected in the CE experiments (data not shown).

^f^ Some HP-NAP co-migrated with the neutral EOF marker, 4-methoxybenzyl alcohol as shown in Fig 5.

### The interaction between HP-NAP purified from gel-filtration chromatography and DEAE resins

**Fig 6:**
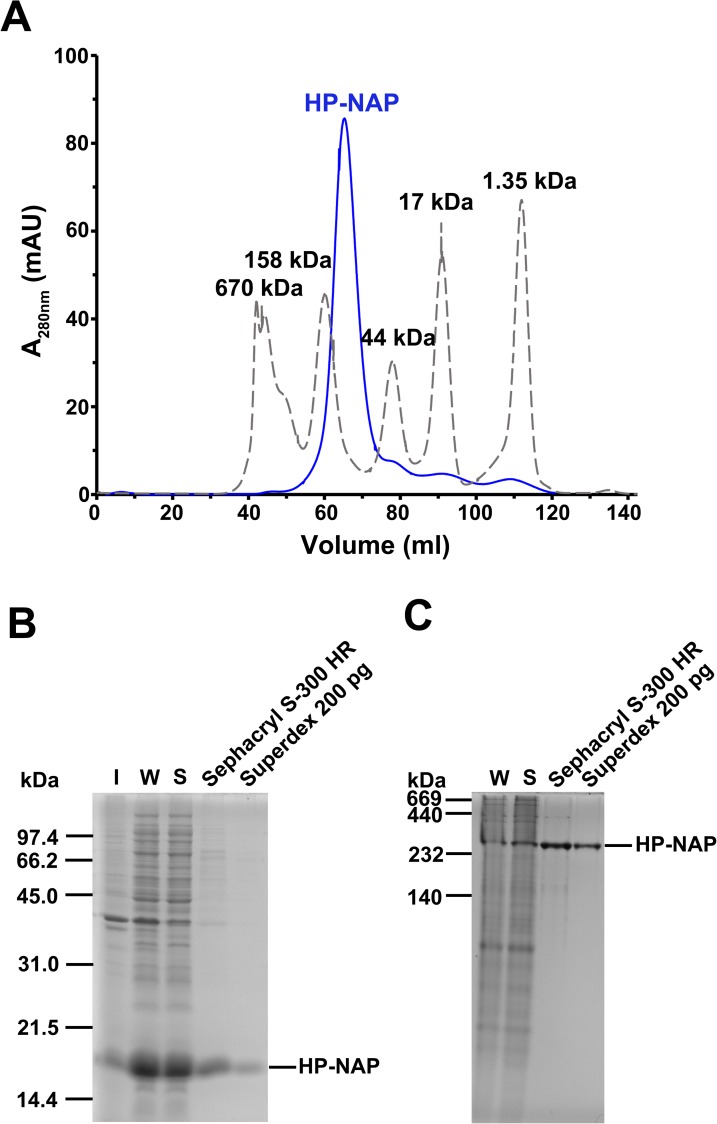
Purification of recombinant HP-NAP expressed in *E*. *coli* by gel-filtration chromatography. HP-NAP expressed in *E*. *coli* BL21(DE3) was purified by two consecutive gel filtration steps using an XK 16/100 column packed with Sephacryl S-300 high resolution resin (Sephacryl S-300 HR) and a HiLoad 16/60 Superdex 200 prep grade (Superdex 200 pg) gel filtration column at pH 9.0 (25°C) as described in Materials and Methods. The chromatogram (**A**) of HP-NAP (━) eluted from Superdex 200 pg was recorded at UV absorbance of 280 nm. The molecular mass of each protein marker (**—-**) was indicated at the top of each peak shown in the chromatogram. Insoluble pellet (I), whole cell lysate (W), soluble cytoplasmic fraction (S), and the eluates from the two columns were analyzed by SDS-PAGE (**B**) and native-PAGE (**C**). Molecular masses (M) in kDa are indicated on the stained gels. Data were representative of three independent experiments.

**Fig 7:**
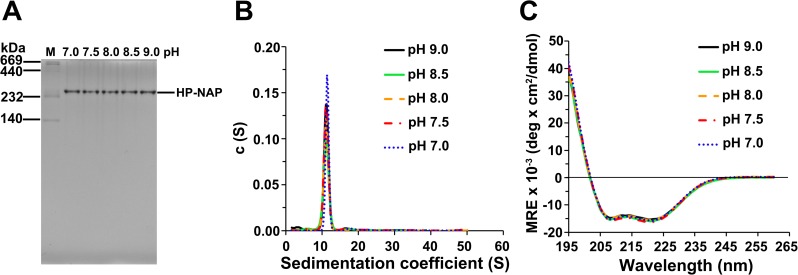
Analysis of molecular and structural properties of HP-NAP purified from gel-filtration chromatography at pH 7.0 to 9.0. HP-NAP purified by gel-filtration chromatography at pH 9.0 (25°C) was adjusted to pH 8.5, 8.0, 7.5, and 7.0 as described in Materials and Methods. (**A**) Native-PAGE analysis of HP-NAP at the indicated pH at 25°C. The molecular masses (M) in kDa are indicated on the stained gels. Data were representative of three independent experiments. (**B**) Sedimentation coefficient of HP-NAP at the indicated pH monitored by analytical ultracentrifugation (AUC) at 25°C. Sedimentation coefficient distribution c(s) was shown as a function of S. The c(s) distribution was analyzed by using the software program SEDFIT. (**C**) The far-UV circular dichroism (CD) spectra of HP-NAP at the indicated pH at 25°C. The spectra were recorded at the wavelength range of 195 to 260 nm at 25°C.

The finding that the majority of recombinant HP-NAP purified from the unbound fraction of DEAE Sepharose resin is neutral at pH 9.0 suggests that the neutral form of HP-NAP might be specifically selected during DEAE ion-exchange chromatographic purification through the collection of the unbound fraction. In order to exclude the charge selectivity that resulted from the ion-exchange chromatography, gel-filtration chromatography was applied to purify recombinant HP-NAP expressed in *E*. *coli* to investigate whether the recombinant HP-NAP purified by these two chromatographic methods behaves differently in terms of their ability to bind DEAE resins and their surface charge status at pH 7.0 to 9.0. The recombinant HP-NAP obtained from gel-filtration chromatography at pH 9.0 eluted as a single peak in the chromatogram (Fig 6A) and its purity was higher than 98% as analyzed by SDS-PAGE (Fig 6B). Native-PAGE analysis showed that the purified HP-NAP was oligomeric with apparent molecular mass around 232 kDa (Fig 6C). The recombinant HP-NAP with a pH adjustment from pH 9.0 to pH 7.0 retained its oligomeric state as analyzed by native-PAGE (Fig 7A). Analytical ultracentrifugation (AUC) was further applied to examine the oligomeric structure of recombinant HP-NAP. At pH ranging from 9.0 to 7.0, HP-NAP sedimented as a major peak, centered at the sedimentation coefficients of 11.1 to 11.4 S (Fig 7B). These results reveal no major change of the multimeric association of recombinant HP-NAP at different pH values investigated. In addition, there is no pH-dependent variation in the α-helix structure of recombinant HP-NAP with a pH adjustment from pH 9.0 to pH 7.0 as analyzed by far-UV circular dichroism (CD) spectroscopy (Fig 7C). Thus, the overall structure of HP-NAP is consistently preserved at all pH values investigated.

**Fig 8:**
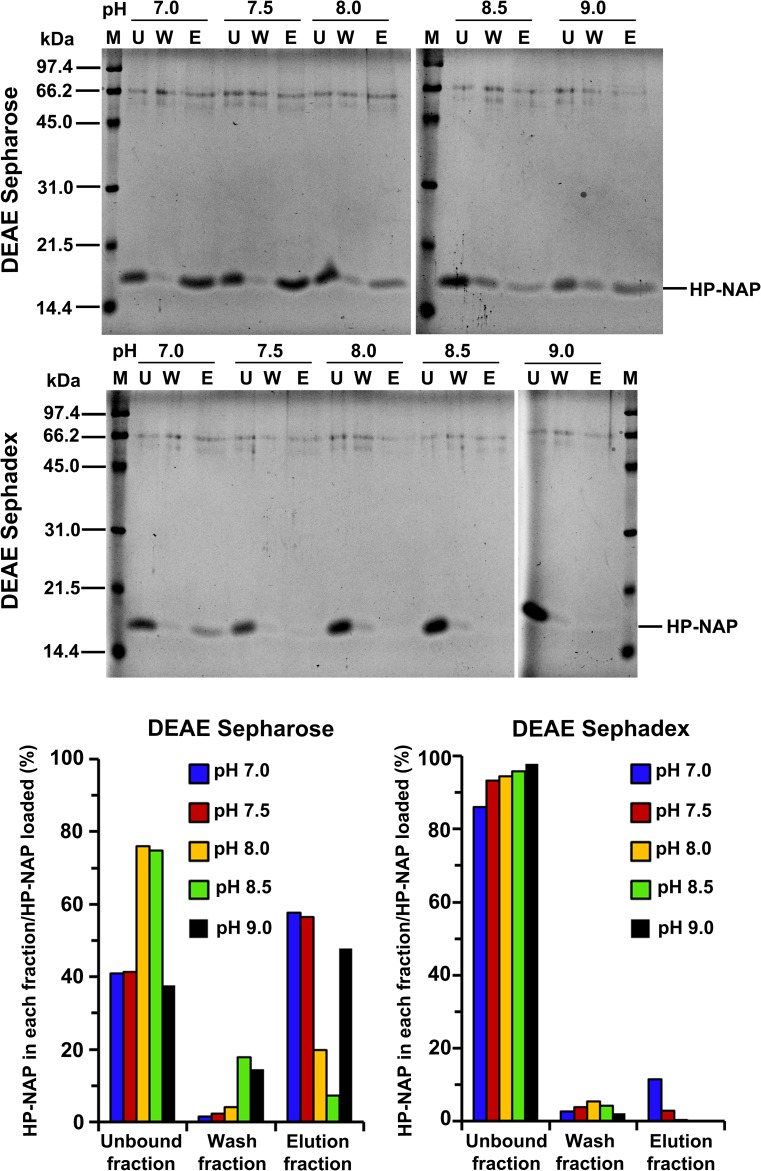
Binding ability of recombinant HP-NAP purified from gel-filtration chromatography to DEAE resins at pH 7.0 to 9.0. HP-NAP purified by gel-filtration chromatography at pH 9.0 (25°C) was kept at pH 9.0 or adjusted to pH 8.5, 8.0, 7.5, and 7.0 and then diluted to a protein concentration of 0.3 mg/ml. The samples were then subjected to batch chromatography by using DEAE Sepharose and DEAE Sephadex resins at their respective pH at 25°C. The unbound (U), wash (W) and elution (E) fractions were analyzed by SDS-PAGE on 15% gels. Molecular masses (M) in kDa are indicated on the stained gels. The percent ratio of the amount of recombinant HP-NAP detected in each fraction to the amount of HP-NAP loaded on the resin at each pH was calculated from the intensity of HP-NAP band on SDS gels for each fraction divided by the sum of those for the unbound, wash and elution fractions. Similar results were obtained from two independent experiments.

The same experimental approaches shown in Fig 3 were applied to HP-NAP purified from gel-filtration chromatography to determine its ability to bind DEAE resins and their surface charge status. The recombinant HP-NAP purified from gel-filtration chromatography at pH 9.0 and those with a pH adjustment from pH 9.0 to pH 8.5, 8.0, 7.5 and 7.0 were first incubated with DEAE resins at their respective pH to examine whether they bind to DEAE resins at the pH values investigated. For DEAE Sepharose resin, more than half of the amount of recombinant HP-NAP was detected in the elution fraction at pH 7.0 and 7.5 (Fig 8). The amount of recombinant HP-NAP detected in the elution was decreased as the pH was increased from 7.0 to 8.5 and then increased as the pH was increased from 8.5 to 9.0 (Fig 8). For DEAE Sephadex resin, only a small amount of recombinant HP-NAP at pH 7.0 was detected in the elution fraction, and the recombinant HP-NAP was mainly present in the unbound fractions at the pH from 7.5 to 9.0 (Fig 8). The minimal binding of pure HP-NAP to DEAE Sephadex resin at pH 7.0 further supports the finding that impure proteins did play a role in helping the recombinant HP-NAP bind to DEAE Sephadex resin at pH 7.0 as shown in Fig 1. At all the pH values investigated, HP-NAP was observed to bind stronger to DEAE Sepharose resin than to DEAE Sephadex resin. The finding also suggests that the negatively charged molecular species of HP-NAP may exist at pH ranging from 7.0 to 9.0.

**Fig 9:**
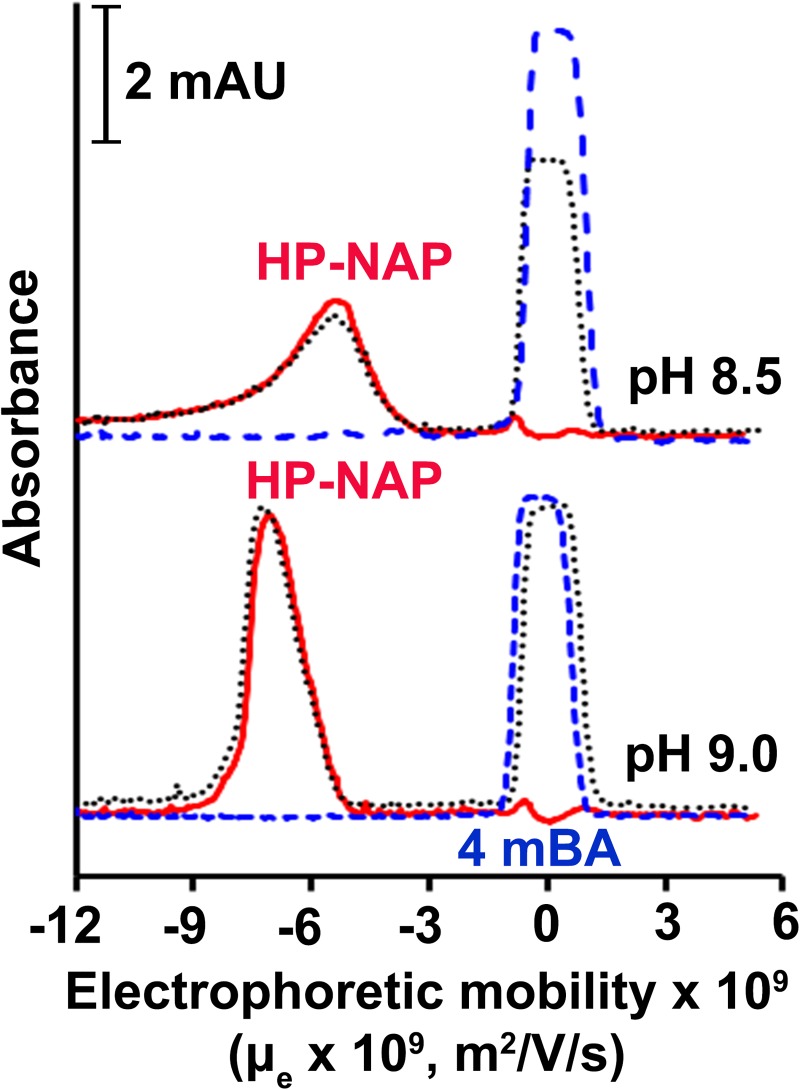
Capillary electropherograms of HP-NAP purified by gel-filtration chromatography at pH 8.5 and 9.0. HP-NAP purified by gel-filtration chromatography at pH 9.0 (25°C) was kept at pH 9.0 or adjusted to pH 8.5 and then diluted to a protein concentration of 0.1 mg/ml. The CE analysis was performed at 25°C with UV absorbance recorded at 220 nm as described in Materials and Methods. The peaks shown in the electropherograms are HP-NAP (━), 4mBA (**—-**), and HP-NAP together with 4mBA (⋯). 4mBA, as the neutral EOF marker, was shown as a peak at μ_e_ = 0 for all electropherograms. Data were representative of two independent experiments.

Capillary electrophoresis was then applied to examine the surface charge of the recombinant HP-NAP purified from gel-filtration chromatography at pH 9.0 and those with a pH adjustment from pH 9.0 to pH 8.5, 8.0, 7.5 and 7.0. Only a single peak corresponding to the molecular species with negative electrophoretic mobility was detected at pH 8.5 and 9.0 (Fig 9). The attempts to obtain the electropherograms of HP-NAP at pH 7.0 to 8.0 were unsuccessful. For gel-filtration purified HP-NAP with a quick and significant pH adjustment from pH 9.0 to pH 8.0, 7.5 and 7.0, it is possible that at the beginning of electrophoresis, the electric field within the capillary induced some irreversible change of these samples and this change led to missing of HP-NAP peaks in the electropherograms. The electrophoretic mobility (μ_e_) of the negatively charged HP-NAP is -5.4 x 10^−9^ and -7.1 x 10^−9^ m^2^V^-1^s^-1^ at pH 8.5 and pH 9.0, respectively (Table 1), indicating that the negative surface charge of recombinant HP-NAP is increased when the pH was raised from 8.5 to 9.0. Even though the electrophoretic mobility (μ_e_) of HP-NAP purified from gel-filtration chromatography is similar to that of recombinant HP-NAP purified by DEAE negative mode chromatography, no neutral charged species was found for HP-NAP purified from gel-filtration chromatography. Thus, recombinant HP-NAP with different charge status can be differentially purified by DEAE negative mode chromatography and gel-filtration chromatography.

## Discussion

In this study, the purification of recombinant HP-NAP with DEAE anion-exchange resin was investigated at pH 7.0 to 9.0 at 25°C. In the purification with DEAE Sepharose resin, HP-NAP was detected in the elution fraction at pH 7.0, 7.5, 8.0 and 9.0, but at pH 8.5, HP-NAP was least detected. In the purification with DEAE Sephadex resin, HP-NAP was detected in the elution fractions only at pH 7.0 and 8.0. However, in the absence of impure proteins, HP-NAP purified from the unbound fractions at pH 7.0 to 9.0 did not bind to DEAE resins at their respective pH except for a very small amount of HP-NAP purified from DEAE Sepharose resin at pH 9.0. By capillary electrophoresis analysis, the surface charge of HP-NAP purified from DEAE negative mode chromatography was neutral at pH 7.0 to 8.0 and either neutral or slightly negative at pH 8.5 and 9.0. However, HP-NAP purified from gel-filtration chromatography was able to bind to DEAE Sepharose resin at pH 7.0 to 9.0 and DEAE Sephadex resin at pH 7.0. At pH 8.5 and 9.0, only the molecular species with negative charge were detected. Thus, recombinant HP-NAP with different charge states can be differentially purified by DEAE negative mode chromatography and gel-filtration chromatography. The finding also suggests that the purification of HP-NAP by DEAE negative mode chromatography is influenced by the surface charge of HP-NAP and the presence of impure proteins.

For ion-exchange chromatography, protein retention on the resin is the result of the electrostatic interactions contributed by the net charge, charge density, and surface charge distribution of the protein, the overall charge and charge density of the resin, and the counter ion concentration and ionic strength of the mobile phase [28, 29]. Previously, we have shown that HP-NAP bound least to DEAE resins at pH 8.0 during the purification at 4°C [23]. In the present study at 25°C, as the pH was increased from 7.0 to 9.0, the negative surface charge of recombinant HP-NAP increased while the positive charge of DEAE resin, a weak anion exchanger, decreased. A minimum binding between HP-NAP and DEAE resin could occur at the pH where their overall charges are too weak for them to interact with each other. This could be a reason why HP-NAP bound least to DEAE Sepharose resin at pH 8.5 during the purification at 25°C. The difference in pH at which HP-NAP bound least to DEAE resins could be explained by the temperature effect on pKa values of the amino acid residues of HP-NAP, the functional group of the resin, and buffer.

The reason why HP-NAP bound weakly to the DEAE Sepharose resin at pH 8.5 during the purification at 25°C could also be resulted from the electrostatic repulsion between HP-NAP and the resin caused by the positive charges present on their surfaces. Unlike *E*. *coli* Dps binding to DNA with its positively charged N-terminus [30], the positively charged protein surface of HP-NAP is mainly responsible for its binding of DNA [31]. Loading of iron to apo-HP-NAP or *E*. *coli* apo-Dps did not influence their abilities to bind DNA as analyzed by gel mobility assay [31], supporting that iron binding did not play a role in modulation of their DNA interaction. In addition, the isoelectric point (pI) of both apo-HP-NAP and the iron-loaded HP-NAP are the same as analyzed by isoeletrofocusing [12], indicating that the surface charge of HP-NAP is not affected by the binding of iron [12]. Furthermore, the abilities of both apo-HP-NAP and the iron-loaded HP-NAP to activate neutrophils are identical [12], suggesting that iron-binding to HP-NAP does not induce the structural changes that cause different interactions with the cell surface of neutrophils. Thus, the charged surface of HP-NAP, rather than its iron binding, is the main factor that plays a significant role in the purification result of DEAE negative mode chromatography. HP-NAP is capable of binding DNA at pH 6.5 to 8.0 [31]. Since the isoelectric point (pI) of HP-NAP is 6.75 [12], the basic residues located in the positively charged patches on the surface of HP-NAP may participate in DNA binding at pH above its pI. Interestingly, a majority of the surface-exposed basic residues of HP-NAP are found to be located at the helix 3 (Leu69-Leu75), the helix 4 (Lys89-Leu114), or the linking coils (His63-Thr68 and Thr76-Ser88), which are critical in stimulating neutrophil activation [19]. These positively charged surface patches could contribute to the electrostatic repulsion between HP-NAP and DEAE resin during the purification. Therefore, the surface charge distribution of recombinant HP-NAP and the overall net charge of the resin at these various pH values might be the reason why HP-NAP bind least to DEAE Sepharose resin at pH 8.5 during the purification at 25°C.

The ability of HP-NAP binding to DEAE Sephadex resin is much weaker than that to DEAE Sepharose resin. HP-NAP does not bind to DEAE Sephadex resin but binds to Sepharose resin at pH 8.5 and 9.0 at 25°C even for the negatively charged HP-NAP purified from gel-filtration chromatography. In addition, capillary electrophoresis showed that the ratio of the peak area of the negatively charged molecular species to that of the neutral molecular species of HP-NAP purified from DEAE negative mode chromatography with DEAE Sepharose resin is much smaller than that with DEAE Sephadex resin. The finding that more negatively charged HP-NAP binding to DEAE Sepharose resin could be due to the different exclusion limits of the two DEAE resins. The exclusion limits of DEAE Sepharose fast flow and DEAE Sephadex A-25 resins used in this study are ~4,000 kDa and ~30 kDa, respectively. DEAE Sepharose fast flow resin has a greater porosity and higher available capacity for larger molecules than DEAE Sephadex A-25 resin. The molecular mass of HP-NAP is ~200 kDa, which falls between the exclusion limits of these two DEAE resins. HP-NAP can only bind on the surface of DEAE Sephadex A-25 resin but enter the pores of DEAE Sepharose fast flow resin. It is probable that at pH 8.5 and 9.0, the surface charge density of Sephadex A-25 resin is not high enough for its interaction with HP-NAP, which carries only a slight negative charge.

Theoretically, HP-NAP is negatively charged above its pI. However, the recombinant HP-NAP obtained from the unbound fractions from DEAE ion-exchange chromatography carried neutral surface charges at pH 7.0 to 8.0 and either neutral or slightly negative surface charges at pH 8.5 and 9.0. Since recombinant HP-NAP with negative surface charge should bind to DEAE resins, it is possible that some negatively charged HP-NAP flowed through the resins at pH 8.5 and 9.0 due to the fact that the binding capacity of the resin is exceeded. The recombinant HP-NAP purified by gel-filtration chromatography, which excludes charge selectivity, was detected in both the unbound and elution fractions at pH 7.0 to 9.0 when subsequently subjected to DEAE Sepharose anion-exchange chromatography. The amount of purified recombinant HP-NAP binding to DEAE Sepharose resin was decreased from pH 7.0 to 8.5 and increased from pH 8.5 to 9.0. The binding of the pure HP-NAP to DEAE Sepharose resin at pH 7.0 to 9.0 strongly indicates that the negatively charged species could exist at these pH values for HP-NAP purified by gel-filtration chromatography. Even though we were not able to determine the charge status of HP-NAP purified by gel-filtration chromatography at pH 7.0 to 8.0 by capillary electrophoresis, only the negatively charged molecular species of recombinant HP-NAP purified by gel-filtration chromatography were found at pH 8.5 and 9.0. This finding is different from the fact that both neutral and negatively charged molecular species of HP-NAP were purified by DEAE anion-exchange chromatography. It is possible that during gel-filtration chromatography, neutral recombinant HP-NAP is easy to aggregate under high protein concentration and then the large aggregates of HP-NAP are removed during the gel-filtration process. As a result, only negatively charged HP-NAP is retained from gel-filtration chromatography.

In conclusion, the surface charge distribution of recombinant HP-NAP and the overall net charge of DEAE resin are the main factors affecting the purification of HP-NAP by DEAE negative mode chromatography. Our findings also reveal that recombinant HP-NAP with different charge status is differentially purified by DEAE ion-exchange negative mode chromatography and gel-filtration chromatography.
